# Microbial Diversity Estimation and Hill Number Calculation Using the Hierarchical Pitman‐Yor Process

**DOI:** 10.1002/sim.70689

**Published:** 2026-08-03

**Authors:** Kevin McGregor, Todd Parsons, Elinor Simons, James Scott, Anita Kozyrskyj, Christopher Quince

**Affiliations:** ^1^ Department of Statistics University of Manitoba Winnipeg Canada; ^2^ Department of Mathematics and Statistics York University North York Canada; ^3^ Department of Epidemiology, Biostatistics, and Occupational Health McGill University Québec Canada; ^4^ Laboratoire de Probabilités, Statistique, et Modélisation Sorbonne Université Paris France; ^5^ Division of Occupational and Environmental Health, Dalla Lana School of Public Health University of Toronto Toronto Canada; ^6^ Pediatrics Department, Faculty of Medicine and Dentistry University of Alberta Edmonton Canada; ^7^ High‐Resolution Microbiomics Group Earlham Institute Norwich UK

**Keywords:** Bayesian non‐parametrics, diversity estimation, hierarchical Pitman‐Yor, Hill numbers, microbiome

## Abstract

**Background:**

The human microbiome comprises the microorganisms that inhabit the various locales of the human body and plays a vital role in human health. The composition of a microbial population is often quantified through measures of species diversity, which summarize the number of species along with their relative abundances into a single value. In a finite microbiome sample, there will be species missing from the target population, which will affect the diversity estimates.

**Methods:**

We employ a model based on the hierarchical Pitman–Yor (HPY) process to model the species abundance distributions over multiple populations. The model parameters are estimated using a Gibbs sampler. We also derive estimates of species diversity, conditional and unconditional on the observed data, as a function of the HPY parameters. Finally, we derive a general formula for the Hill numbers in the HPY context.

**Results:**

We show that the Gibbs sampler for the HPY model performs well in simulations. We also show that the conditional estimates of diversity from the HPY model improve over naïve estimates when species are missing. Similarly, the conditional HPY estimates tend to perform better than the naïve estimates especially when the number of individuals sampled from a population is small. Finally, we illustrate results of applying the HPY model in an infant gut microbiome dataset.

## 1 Introduction

Human microbiome studies attempt to quantify the makeup of the different species that occupy the human body. The composition of the gut microbiome in particular plays an important part in human health. Modelling species abundance distributions in this framework has long been a goal in metagenomic datasets. There have been a number of methods based on Bayesian non‐parametrics developed to model these kinds of community dynamics. In particular, the dependent Dirichlet process has been applied in this context to species abundance distribution and diversity estimation, and has additionally been used to address the effects of covariates on these entities [1, 2]. However, these models were created from a statistical standpoint, and do not reflect the underlying principles of community ecology. In contrast, there are alternative statistical methods that attempt to describe and capture the dynamics of such distributions in a manner that is faithful to principles of ecological theory. Nevertheless, there are competing perspectives on how ecological processes and the environment affect species distributions [3]. For example, one theory suggests that species assemblage is characterized by niches, which are defined by the allocation of resources in the population [4]. There also exists a competing theory that describes “neutral” models, which assume that species are functionally equivalent and that processes such as immigration and birth/death events primarily contribute to community diversity [5]. This theory of neutrality in ecology proposes that most variation in the number and types of species present is a result of chance, as opposed to ecological differences between species. Therefore the birth rate of a particular species depends on the number of individuals of that species present in the population, rather than on the species' ability to survive in the environment. This is analogous to the theory of neutrality for gene alleles that originated in the field of population genetics [6, 7]. One important doctrine in neutral theory is Hubbell's Unified Neutral Theory of Biodiversity [8]. In Hubbell's theory, it is assumed that there are a number of distinct local communities (sometimes called local populations) that are subject to immigration and birth/death processes. Immigration into each community is independent of the other communities, and all individuals that immigrate to a community do so from a conceptual *metacommunity* shared by all local communities. Immigration can happen at different rates across populations. The birth rate in the neutral model is on a *per capita* basis—births are more likely to occur for more highly abundant species. Additionally, the rate of speciation, that is, the frequency at which new species appear (i.e., species that have not yet been observed in *any* population), is a defining parameter of the top‐level metacommunity. Note that the metacommunity and the local communities in Hubbell's model are sometimes referred to in the context of the *mainland‐island* model [9], where immigration occurs from a mainland (analogous to metacommunity) to local islands surrounded by uninhabitable space (analagous to the local communities).

Harris et al. [10] showed that the class of neutral models, when multiple general local communities with differing population dynamics are considered and under conditions on individual mean reproductive success, converges to the hierarchical Dirichlet process (HDP) with large local population size. They developed a Gibbs sampler for the HDP given a matrix of species counts among multiple local communities. The authors also presented a means for testing for neutrality in the populations after fitting the model. However, microbial abundance data has suggested that ranked species abundance distributions follow a power‐law tail [11]. That is, if we consider the ranked proportions of species in a population, $p_{k}$, k=1,2,… such that $p_{1}>p_{2}>p_{3}>\ldots$, then we have the following relationship for the ranked abundances:

$$\begin{array}{l} p_{k}\propto k^{-a} \end{array}$$

for some a>0 [12]. One limitation of the Dirichlet process model is it cannot accommodate a power‐law tail in its species distribution. Conversely, a related process called the *Pitman‐Yor process*, can indeed generate a species distribution that exhibits a power‐law tail [13]. It is then of value to pursue the question as to whether the Pitman‐Yor model is more appropriate for modeling the species frequency distribution than the Dirichlet model. In this article, we investigate the use of the Pitman‐Yor process in a hierarchical formulation called the *hierarchical Pitman‐Yor process* (HPY process) to model abundance data in multiple microbial populations. The HPY model has been recently used in species discovery [14] as well as in single‐cell RNA‐Seq data [15]. However, to the authors' knowledge, this model has not previously been used in a species diversity estimation framework, nor have estimates of diversity metrics been derived in terms of the HPY model parameters. Empirical estimators for species diversity will always be confounded by finite sample sizes.

Therefore, in this article we provide such a derivation of diversity metrics in this context; in particular, we derive a general formula for the *Hill numbers* as a function of HPY parameters. We develop a Gibbs sampler to fit the model parameters. In addition to the Gibbs sampler, we derive expressions for two measures of species diversity in the context of the HPY process. We perform an extensive simulation study to investigate the performance of the HPY model under different situations, as well as the efficacy of the measures of species diversity defined in the HPY context. As such, the objectives of this work are as follows:

1. To evaluate the appropriateness of the HPY model over the HDP model in an infant gut microbiome study, in particular, in the “tail” of the ranked species abundance curve.
2. To explore the possibilities of estimating both alpha‐ and beta‐diversity metrics using the HDP and HPY models and compare to empirical estimates.

### 1.1 Infant Gut Microbiome Study

The motivating study for this work comes from [16], who investigated whether proximity to natural environments influences the diversity of the gut microbiota in infants. The study measured the gut microbiota using 16S sequencing on fecal samples from infants at 4 months of age. Native vegetation data was collected from the Urban Primary Land and Vegetation Inventory (uPLVI) from Edmonton, Alberta. Analyses were stratified by sex, breastfeeding status, and presence of household pets. In total, there were 355 infant fecal samples collected, all of which came from the Canadian Healthy Infant Longitudinal Development (CHILD) cohort.

While the focus of the original publication was alpha diversity, it should be noted that all microbiota samples were rarefied. Rarefying can be troublesome in diversity analyses as data from higher read‐depth samples are discarded [17]. Therefore, in this article, we analyze diversity in the intestinal microbiota using estimates from the HPY model, which avoids rarefying the data. We also obtained several covariate measures for each infant, including sex (170 female, 172 male), breastfeeding status (63 no breastfeeding, 99 partial breastfeeding, 180 exclusive breastfeeding), and presence of household pets (171 yes, 171 no). We were not able to access the vegetation data as it is stored in the repository of another agency (uPLVI) and therefore was not available through the CHILD study portal.

We begin in the next subsection with the definition of the Pitman‐Yor Process. In the Methods section (Section 2.1), we introduce the hierarchical Pitman‐Yor model and give details for the Gibbs sampler as well as a description of calculating diversity in this model. In Section 3 we present results for an extensive simulation study that investigates the performance of the HPY model. In Section 4 we show the results of applying the HPY and HDP models on the infant gut dataset.

### 1.2 Pitman‐Yor Process

Assume that, for 0≤α<1 and γ>−α, we generate a sequence of random variables $V_{k}$, for k=1,2,… such that $V_{k}∼\text{Beta}(1-\alpha ,\gamma +k\alpha )$. Furthermore, define the following:

(1) $$p_{1}=V_{1},\;p_{k}=(1-V_{1})\ldots (1-V_{k-1})V_{k},\;\text{for}\;k\geq 2.$$

Then the two‐parameter *Griffiths, Engen, and McCloskey distribution* is defined as the joint distribution of $\left(p_{1},p_{2},\ldots \;\right)$ and is abbreviated as GEM(γ,α) [18].

Let $X_{k}^{*}$, k=1,2,… be a sequence of independent samples from some distribution H. Some examples for H could include a uniform distribution over a finite number of categories, a Gaussian distribution, or even a GEM distribution. If we draw the vector p∼GEM(γ,α) independently from each $X_{k}^{*}$, then the Pitman‐Yor process (or two‐parameter Poisson‐Dirichlet process) is defined as

(2) $$\begin{array}{l} \overset{\infty }{\underset{k=1}{\sum }}p_{k}\delta_{X_{k}^{*}}, \end{array}$$

where $\delta_{X_{k}^{*}}$ is a discrete measure at $X_{k}^{*}$ [19]. The Pitman‐Yor process is abbreviated as PY(α,γ,H). α and γ are called the *discount* and *concentration* parameters, respectively. H is referred to as the *base distribution*. The Dirichlet process is a special case of the Pitman‐Yor process where the discount parameter α=0.

A convenient way of drawing from the Pitman‐Yor process is through the *Chinese restaurant process* representation. Assume that we have already drawn $X_{1},\ldots ,X_{n}$ among which we have drawn the values $X_{1}^{*},\ldots ,X_{K}^{*}$ directly from the base distribution H, for some 1≤K≤n. If H is continuous, then all of $X_{1}^{*},\ldots ,X_{K}^{*}$ are distinct; however, if H is discrete, then some of the values may not be distinct. Then the distribution of $X_{n+1}$ conditional on $X_{1},\ldots ,X_{n}$ is:

(3) $$\begin{array}{l} X_{n+1}|X_{1},\ldots ,X_{n},\gamma ,\alpha ,H∼\overset{K}{\underset{k=1}{\sum }}\frac{n_{k}-\alpha }{\gamma +n}\delta_{X_{k}^{*}}+\frac{\gamma +K\alpha }{\gamma +n}H, \end{array}$$

where $n_{k}$ represents the number of times the value $X_{k}^{*}$ appears among $X_{1},\ldots ,X_{n}$. More simply, $X_{n+1}$ is sampled either from an existing value $X_{k}^{*}$ with probability proportional to $n_{k}$, or from the base distribution H. The values $X_{1}^{*},\ldots ,X_{K}^{*}$ are called “tables” in the Chinese restaurant configuration. The tables may also be referred to simply by their indices 1,…,K.

### 1.3 Diversity

One of the most important descriptive tools in microbial community estimation is species diversity. Diversity aims to create a quantitative description of a population that incorporates information such as the total number of species in a population and the relative frequencies of those species. For example, one commonly applied measure of diversity is Simpson's index, which concerns the probability of sampling the same species twice from a population. Alternatively, the Gini–Simpson index is calculated as the probability of sampling two different species in subsequent draws (i.e., the complement of the above definition). The latter definition is used in this article. Assume a population contains K distinct species and that the proportion of species k is represented by $p_{k}$. Then the Gini–Simpson index, denoted by D, is then written as [20]:

(4) $$\begin{array}{ll} D & =1-\overset{K}{\underset{k=1}{\sum }}p_{k}^{2}. \end{array}$$

A larger value of D implies the probability of obtaining the same species in two subsequent samples from the population is small. Thus, the closer the value of D is to one, the more diverse the population under consideration.

The fundamental problem in estimating diversity is that it is unlikely that a sample from a population will include all K species that exist in the population. To illustrate the problem, consider a population with three species (K=3), where the counts of the three species are $\left(n_{1},n_{2},n_{3})=(5,4,1\right)$. If we calculate the Gini–Simpson index naïvely based on Equation (4) we get D=0.58. Now, consider a sample from this population where we obtain the counts $\left(n_{1},n_{2},n_{3})=(3,2,0\right)$. Calculating the Gini–Simpson index on the sample proportions gives D=0.48. Thus, the diversity in the population has been underestimated, simply because we failed to sample individuals from species 3.

Model assumptions can help to estimate species diversity in the presence of missing species. Methods for estimating the Gini–Simpson index in the mainland–island model assumed in Hubbell's neutral theory of biodiversity have been developed by [21]. Expressions to estimate the Gini–Simpson index have not yet been developed in the HPY context, however. Thus, in this article we propose the derivation of an expression of the Gini–Simpson index in this context. The idea is that the HPY Gibbs sampler proposed in this article could be applied to species abundance data, and estimates of the HPY model parameters could be obtained. From there, the Gini–Simpson index could be calculated using the estimated HPY model parameters. The advantage of this approach is that, even if a particular population has missing species, the model structure assumed in the HPY framework can help to model the probability of discovering a new species (i.e., one that has not yet been observed). There is also a sense of “borrowing strength” between populations, in that we can account for sampling probabilities for a species that has a zero count in a population by considering the frequencies of that species in another population.

The framework that we pursue to estimate the Gini–Simpson index using the HPY model depends on the calculation of the *Hill numbers* [22], which are defined for integers q≥2 as

(5)

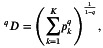


so that the Gini–Simpson index as defined in Equation (4) can be written as 

(6)

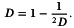


In this article, we show that the Chinese restaurant franchise representation of the HPY process admits a convenient way to derive the reciprocal of 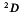. Additionally, we provide a formula for 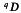 for general q≥2 in the HPY context.

## 2 Methods

In this section, we outline the model for the HPY process and describe the Gibbs sampler used to fit the model. Additionally, we present a formula for the Gini–Simpson index in the context of the HPY model parameters. In the mainland–island model, each individual in a local community can be traced back to an ancestor that immigrated to the community. In the hierarchical Pitman‐Yor model, the “tables” in the Chinese restaurant representation of the Pitman‐Yor process are analogous to individuals' ancestors that immigrated in the mainland‐island model. Multiple individuals in a community could have descended from the same ancestor, and there can be multiple ancestors of the same species. The makeup of the ancestors is not directly observable and is therefore treated as latent variables in this framework. In this article, we use the following notation:

- J is the number of observed local communities
- K is the total number of observed species across all local communities
- $Y_{jk}$ is the observed count of species k in population j
- $t_{jp}$ is the ancestor (“table”) from which individual p in community j descends
- $m_{jk}$ is the number of ancestors in community j corresponding to species k
- $n_{jtk}$ is the number of observations in community j that descended from ancestor t, corresponding to species k.
- $k_{jp}$ is the species of individual p in community j (a number from 1 to K)
- $\psi_{jt}$ is the species of the $t^{th}$ ancestor in community j (a number from 1 to K)
- $A_{jt}$ is the label for the $t^{th}$ ancestor in community j.

We use the symbol · in a subscript to denote the summation of all values over a particular dimension. For example, $m_{j\cdot }=\sum_{k=1}^{K}m_{jk}$ is the number of ancestors in population j over all species k=1,…,K.

### 2.1 Hierarchical Pitman‐Yor Process Model

**FIGURE 1:**
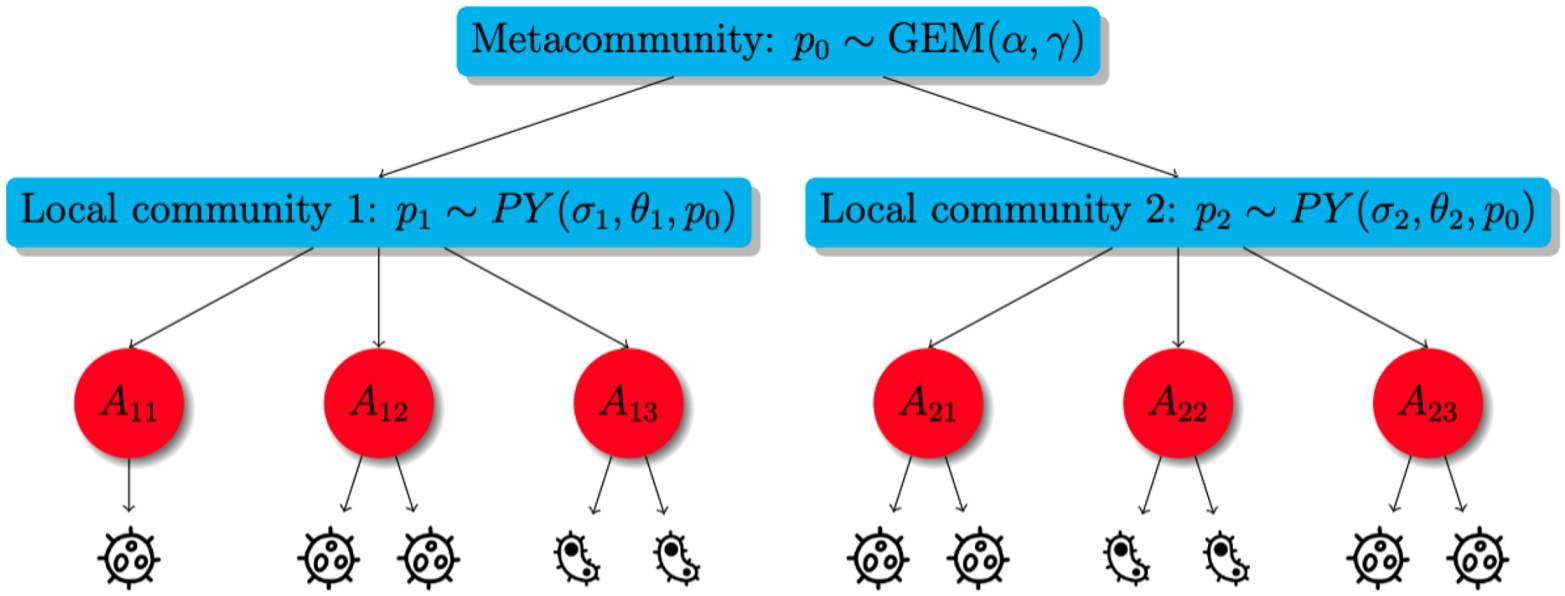
Schematic showing the HPY model assuming two local populations. A red node labelled $A_{jt}$ corresponds to the $t^{th}$ ancestor in local population j. The microbes pictured at the bottom correspond to descendants of the ancestors; that is, the microbes present in the local communities. The observed species abundance table is a sample from the descendant microbes.

In this section, we introduce the hierarchical Pitman‐Yor process and describe how it is connected to the mainland‐island model. The model is defined as follows:

$$\begin{array}{ll} p_{0}|\alpha ,\gamma & ∼\text{GEM}(\alpha ,\gamma )\\ p_{j}|\sigma_{j},\theta_{j},p_{0} & ∼\text{PY}(\sigma_{j},\theta_{j},p_{0})\\ Y_{j}|p_{j} & ∼\text{Multinom}(n_{j\cdot \cdot },p_{j}), \end{array}$$

for j=1,…,J. The vector of proportions $p_{0}$ represents the abundance of species in the metacommunity, which is assumed to follow a GEM distribution. Each vector $p_{j}$ represents the abundance distribution of local population j, and is assumed to follow a Pitman‐Yor process with base distribution $p_{0}$. Therefore, sampling a new ancestor in local community j is akin to sampling from the abundance distribution $p_{0}$ of the metacommunity. Finally, the vector of observed species counts in population j, denoted by $Y_{j}$, is sampled from a multinomial distribution with its proportion vector set to $p_{j}$. This final step represents the sampling procedure from the local community (i.e., sequencing a microbe in a 16S or metagenomic study). The HDP developed in [10], was shown to be the large population size limiting distribution for Hubbell's neutral model under certain conditions on individual mean reproductive success. In the hierarchical Pitman–Yor (HPY) model, we include the discount parameters α and $\sigma_{j}$; a nonzero value for the discount parameter corresponds to a departure from the HDP; that is, the conditions on the moments in Harris et al. [10] are not satisfied. The discount parameter can be used as a measure of departure from neutrality [23]. Figure 1 gives a schematic of the assumed HPY model assuming two local populations.

Priors for the top‐ and local‐level discount and concentration parameters also must be specified in the HPY model:

$$\begin{array}{ll} \alpha & ∼\text{Uniform}(0,1)\\ \sigma_{j} & ∼\text{Uniform}(0,1)\\ \gamma & ∼\text{Gamma}(a_{0},b_{0})\\ \theta_{j} & ∼\text{Gamma}(a_{j},b_{j}) \end{array}$$

for j=1,…,J. Here, $a_{0}$ and $b_{0}$ are respectively the shape and scale hyperparameters of the gamma distribution for the top‐level concentration parameter γ. Likewise, $a_{j}$ and $b_{j}$ are the shape and scale hyperparameters in the prior for the local‐level concentration parameter $\theta_{j}$.

### 2.2 Diversity Estimation in the HPY Framework

In this article, we consider the Gini–Simpson index in the context of the HPY model. The idea is, rather than calculating the Hill number reciprocal 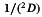 directly from the observed proportions, we can infer in a given population the probability of sampling the same species twice in a row based on the sampling probabilities prescribed by the HPY process. We can do this by considering the Chinese restaurant process representation of the Pitman–Yor process. That is, when a particular species is sampled in a population, the probability of observing that same species a second time is slightly altered, since the corresponding ancestral counts will have been modified. Thus, the joint probability of sampling the same species twice can be calculated by considering all possible configurations of sampling the same species twice. We consider two forms of the expression for the Gini–Simpson index: the first form, which we call the *unconditional* Gini–Simpson index, uses only the discount and concentration parameters from the HPY model.

Theorem 1 *The unconditional Gini–Simpson index for population* j, *defined as a function of* 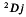, *is written in terms of the HPY parameters as*

(7)

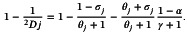


*The proof is given in Section S*2.1.1.

The second form of the Gini–Simpson index, which we call the *conditional* Gini–Simpson index, uses the HPY parameters as well as the observed species counts and ancestral states $t_{jp}$. The unconditional index represents the overall probability of sampling the same species twice in the HPY framework, and the conditional index represents the probability that the *next two* samples are of the same species, given a particular number of individuals have already been sampled.

Theorem 2 *The conditional Gini–Simpson index for population* j *is written in terms of the HPY parameters and the observed counts and ancestor configurations as*

(8)

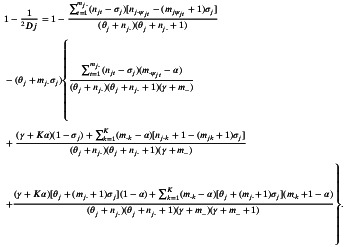

*The proof is given in Section S*2.1.2.

Considering the error in estimating the HPY discount and concentration parameters, we expect that additional leveraging of the observed counts and estimated ancestral states will aid in estimation accuracy.

The Gini–Simpson index is a measure of alpha diversity—in other words, it measures the amount of diversity in a single population. In this article, we also consider the equivalent probability calculation across populations, that is, the probability of sampling one species in one population and the same species in another population. This could be applied as a measure of beta diversity, which measures the discrepancies in species abundance distributions between populations. That is, if the species proportion vector for individual i is $p_{i}={\left(p_{i1},\ldots ,p_{iK}\right)}^{⊤}$, then the beta‐diversity version of Simpson's index between populations i and j would be given by $\sum_{k=1}^{K}p_{ik}p_{jk}$. This probability can similarly be calculated in the context of the hierarchical Pitman‐Yor model. We have derived expressions for the beta‐diversity version of Simpson's index (conditional and unconditional).

Theorem 3 *The unconditional beta‐diversity Simpson's index between populations* j *and* $j^{\prime }$ *is written in terms of the HPY parameters as*

(9)

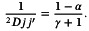

The unconditional beta‐diversity Simpson's index is the probability that two individuals, each taken from different, previously unsampled sites j and $j^{\prime }$, are of the same species. *Absent any prior information about* j *and* $j^{\prime }$, that is simply the probability that a species from the metacommunity is present at two sites. Likewise, we have derived the conditional form of beta‐diversity Simpson's index.

Theorem 4 *The conditional beta‐diversity Simpson's index between populations* j *and* $j^{\prime }$ *is written in terms of the HPY parameters and the observed counts and ancestor configurations as*

(10)

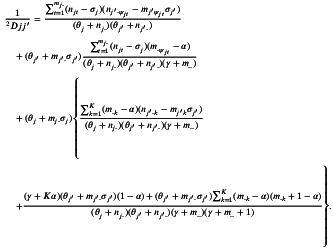

Details for beta‐diversity results can be found in Section S2.2. Since this quantity does not have a natural scale, one can transform it in the following way:

$$M_{ij}=\frac{2\sum_{k=1}^{K}p_{ik}p_{jk}}{\sum_{k=1}^{K}p_{ik}^{2}+\sum_{k=1}^{K}p_{jk}^{2}}$$

This quantity is bounded between 0 and 1, and corresponds to Morisita's overlap index [24]. In the practical applications in this article, we use $M_{ij}$ as a measure of beta diversity. The denominator sums are simply the alpha‐diversity versions of Simpson's index (i.e., the complement of the Gini–Simpson index) in populations i and j, respectively.

Finally, we have derived a representation for (unconditional) Hill numbers for any integer q≥2.

Theorem 5 *The unconditional Hill number in population* j, *denoted by* 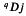, *can be written in terms of the HPY parameters as*

(11)

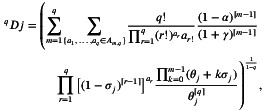


*where* $A_{m,q}=\{\{a_{1},\ldots ,a_{q}\}:a_{1}+\cdots +a_{q}=m,a_{1}+2a_{2}+\cdots +qa_{q}=q\}$.

Though this theorem holds for any integer q≥2, we only numerically explore the q=2 case in this work. The proof of Theorem 5 is given in Section S2.1.1.

### 2.3 Gibbs Sampler for the HPY Model

In order to fit the HPY model defined in Section 2.1, we use a Gibbs sampler. The parameters that must be updated in each step of the Gibbs sampler are the top‐level GEM parameters α and γ; the local‐level PY parameters $\sigma_{j}$ and $\theta_{j}$, for j=1,…,J; and the ancestor indicators $t_{jp}$ for each j=1,…,J and $p=1,\ldots ,n_{j\cdot \cdot }$.

As the local‐level abundances are represented by a Pitman‐Yor process, updates for the parameters $\sigma_{j}$ and $\theta_{j}$ can be obtained by slice‐sampling procedures defined by [25]. This algorithm is outlined in Algorithm S1 in the supplement. Note that the full conditionals for the concentration parameters are not log‐concave. Buntine uses the technique from [26] to generate an auxiliary beta‐distributed random variable. The joint distribution of the concentration parameter and the auxiliary variable is log‐concave (see Section S1.2.2), which allows the use of a slice‐sampler. At the top level, we have a GEM distribution; however, the slice samplers from Buntine can still be applied with a slight modification to the table count vectors. Details on these procedures are given in Section S1.1 for the discount parameters, and Section S1.2 for the concentration parameters. For the ancestral indicators $t_{jp}$, j=1,…,J and $p=1,\ldots ,n_{j\cdot \cdot }$, we apply the method used in [14]. In their algorithm, each individual (i.e., each organism) is removed from the population and reassigned to a new or existing ancestor with probabilities defined by the Chinese restaurant process. Details of that procedure are outlined in Section S1.3. Algorithm 1 outlines a single iteration of the Gibbs sampler used to fit the model.

ALGORITHM 1 **Iteration i of the HPY sampler.**

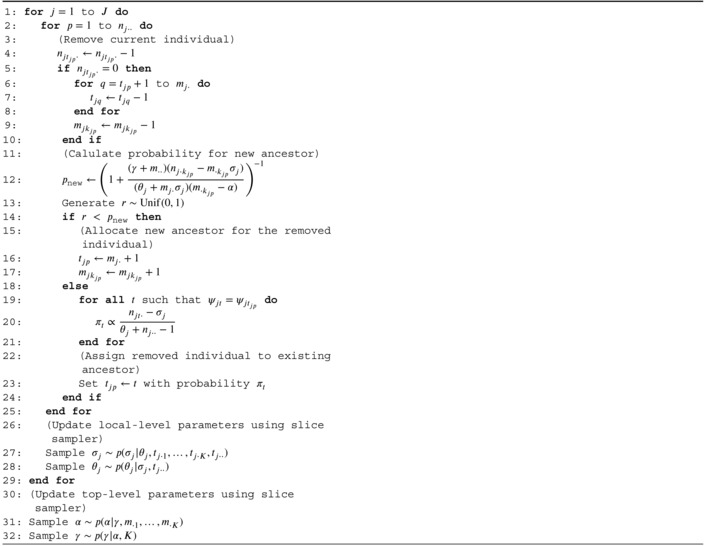

It is not immediately obvious what choices would be best for the hyperparameters $a_{0}$, $b_{0}$, $a_{j}$, and $b_{j}$. To guarantee the log‐concavity of the joint distribution of the concentration parameters, we need to choose the shape parameters so that $a_{0}\geq 1$ and each $a_{j}\geq 1$ (see Section S1.2.2). We find that setting these shape parameters to 1.1 works well in practice. The sampler is fairly sensitive to the choice of the scale parameters $b_{0}$ and $b_{j}$. However, we do find in practice that setting all the scale parameters equal to the number of observed species K leads to good estimation accuracy. This allows the prior means for the concentration parameters to scale as a function of the number of observed species, which is necessary since we have observed that a poorly specified setting for this prior mean can negatively affect the posterior distributions of these parameters. This configuration corresponds to the recommendation in Buntine's libstb C library, which is used to sample the Pitman‐Yor parameters [25].

### 2.4 Simulation Study

To validate the Gibbs sampler for the HPY model, we have designed an extensive simulation study. We simulate data from the hierarchical Pitman‐Yor process using its Chinese restaurant process representation. That is, for every new individual sampled in local population j, we will perform one of three options (1) sample from an existing ancestor, thus assigning that individual the same species as the ancestor; (2) sample from an existing species at the top level; or (3) sample a new species at the top level.

In the simulation study, we simulate data under multiple realistic configurations of the parameters in the HPY model. For the local‐level parameters, we consider the values $\sigma_{j}\in \{0.2,0.5\}$ and $\theta_{j}\in \{5,25\}$. For the top‐level parameters, we consider the values α∈{0.2,0.5,0.8} and γ∈{5,25,50}. We generate 10 local communities, and assume the same values for the local parameters across all communities. In all cases, the total number of individuals generated from the HPY process is 100 000 in each community; however, the final number of individuals sampled in each community in the multinomial step is $n_{j\cdot \cdot }\in \{500,1000,5000\}$ (i.e., this is the number of individuals that would be observed in the species abundance vector $Y_{j}$ in each population j). We generated 50 replications of each simulation scenario. For each of the simulation replications, the HPY Gibbs sampler was run for 2000 steps along with 1000 burn‐in steps. To obtain parameter estimates, we take the posterior mode of the MCMC samples. The posterior mode is more appropriate than the posterior mean in this context as the posterior distributions of the concentration and discount parameters are usually quite skewed due to the constraints on their supports.

Results from the simulation study are shown in Section 3. We examine the accuracy of the estimated parameters by comparing the posterior mode of the parameter samples from the Gibbs sampler to the true simulated values. We also calculate the Gini–Simpson index to measure alpha diversity in each population (see Equations (S.13) and (S.14)) and beta diversity to compare across populations (see Equations (S.15) and (S.16)); we do this for both the unconditional and conditional versions of the Gini–Simpson index. We compare the Gini–Simpson index from the HPY model to the naïvely calculated indices, that is, summing the squared observed proportions in each sample.

We perform an additional simulation study to investigate the stability of alpha‐diversity estimates when adding new local populations to the HPY sampler. We run an additional experiment where an initial set of J=10 local populations is taken from the HPY process and Simpson's index is calculated using estimates our Gibbs sampler. Then, subsequent populations are added to the dataset, and the Gibbs sampler is rerun including the new populations. Simpson's index is recalculated on the initial 10 local populations. We repeat this procedure for J∈{10,20,40,60,80,100} and plot the resulting Simpson's index in the initial 10 populations. We do this process for a set of homogeneous populations ($\theta_{j}=5,\sigma_{j}=0.5$ for all j), and again for a set of heterogeneous populations ($\theta_{j}∼\text{Unif}(1,10)$ and $\sigma_{j}∼\text{Unif}(0.1,0.5)$).

To check whether alpha diversity is comparable between metacommunities, we run an experiment where we simulate 20 local populations across two separate metacommunities (10 populations in each). The HPY parameters are the same in the two metacommunities: (γ=5, α=0.3) and ($\theta_{j}=5,\sigma_{j}=0.5$ for all j). After simulating all the local populations, random subsets are taken (i.e., multinomial step) and the HPY Gibbs sampler is run separately for both metacommunities. We compare rankings in the results section.

### 2.5 Data Analysis in Infant Gut Microbiome Study

In this article, we wish to estimate diversity in the infant gut microbiome study, using the HPY model and naïvely, and compare diversity within the various covariate groups. The available covariates are pets at home (yes/no), breastfeeding status (none/partial/exclusive), and sex. We also explore some technical details such as the appropriateness of the fitted HPY model to the observed data and the effect of sequencing depth on the diversity estimates.

## 3 Simulation Results

In this section we outline the results of the simulation study described in Section 2.4. First we consider the estimation accuracy of the various parameters in the HPY model (γ, α, $\theta_{j}$, $\sigma_{j}$). Results from these investigations are shown in Section S3. Each of these plots shows estimation errors as the difference between the estimated parameter (posterior mode) and the true simulated value. These errors are normalized by dividing by the true simulated values to facilitate comparisons across simulation scenarios.

In Figure S1, we consider the normalized errors from estimating the top‐level concentration parameter γ. Estimates generally appear to be unbiased. There is little improvement in estimation accuracy with increasing sample size in the local populations. Conversely, there does appear to be better estimation accuracy when the true value of the top‐level discount parameter (α) is smaller. Higher values of α correspond to a heavier tail when considering the species abundance distribution aggregated across populations. That is, a higher top‐level discount parameter implies many singleton species, which could contribute to the difficulties in estimation accuracy in this particular parameter configuration.

Simulation results for the top‐level discount parameter α are shown in Figure S2. Again, the estimates do not appear to have any particular bias. In many cases there does appear to be a slight improvement in estimation accuracy with increasing sample size. There is a substantial increase in estimation accuracy for larger values of the true simulated value of α. In the $n_{j\cdot \cdot }=500$ configuration, there is generally better estimation accuracy for $\sigma_{j}=0.2$ than for $\sigma_{j}=0.5$.

In Figure S3, we show the estimation accuracy in simulations for the local‐level concentration parameters $\theta_{j}$, j∈{1,…,J}. There is generally better estimation accuracy when the true value of the local concentration parameters is $\theta_{j}=5$ compared to $\theta_{j}=25$. It also seems that there is a slight positive bias in estimates for this parameter, though the reason for this potential bias is still unclear. Similarly in Figure S4 we show results for the local‐level concentration parameters $\sigma_{j}$, j∈{1,…,J}. In most cases there is a noticeable improvement with increasing sample size. In some cases there is an improvement for θ=25 as opposed to θ=5, however, this improvement is not consistent across all simulation scenarios. There also appears to be a slight negative bias for these parameters. In the MCMC samples there is typically a strong negative correlation between the local concentration and discount parameters due to the fact that both parameters are related to the total number of observed species. This could explain why the biases for the local concentrations are of the opposite sign from the biases of the local discount parameters.

Next, we investigate the results of estimating the Gini–Simpson's index using the HPY and HDP models. First, the unconditional and conditional versions of Gini–Simpson's index are calculated from Equations (7) and (8), respectively, using MCMC samples from the HPY model. The HDP conditional estimates can be calculated similarly (i.e., all discount parameters are 0 in that case). We calculate each index for each of the 2000 MCMC samples from the Gibbs sampler. Then, the final estimate for each version of the Gini–Simpson index is taken as the posterior mode. We compare the index values from the HPY and HDP models to a naïvely calculated index from the observed proportions in each sub‐sampled population. The true value is considered to be the result of applying Equation (4) to the proportions in the full simulated HPY dataset (which would be unobserved in practice).

**FIGURE 2:**
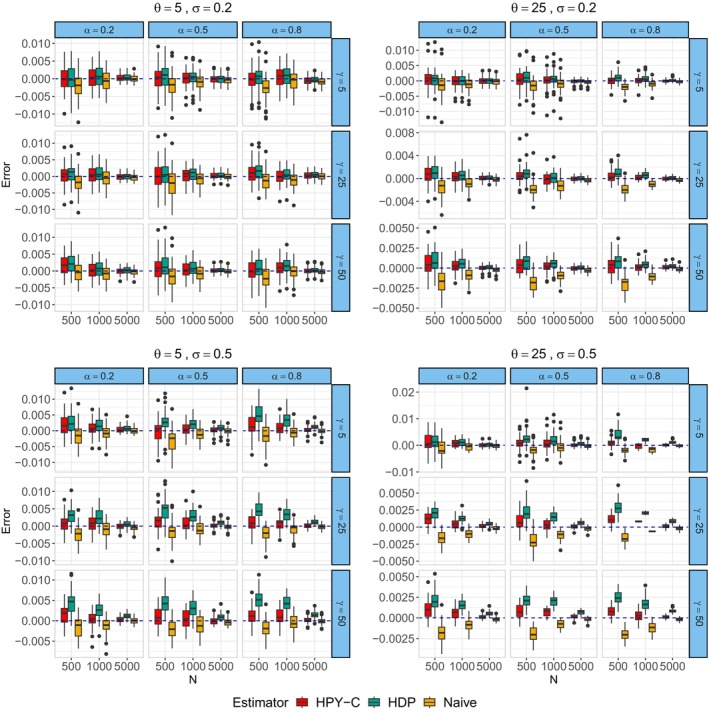
Estimation error for the Gini–Simpson index comparing the conditional index from the HPY model (HPY‐C), the Gini–Simpson index from the HDP model (HDP), and naïve estimates in simulated data.

In Figure 2 we compare the errors for the conditional and naïve estimation methods; the unconditional version also appears in Figure S5, but performs significantly worse than other methods. There is consistently greater variation in the amount of error for the unconditional Gini–Simpson index. For the sake of more easily making a comparison between conditional and naïve estimates, we have omitted the unconditional from Figure 2. The naïvely estimated Gini‐Simpson's index is often underestimated, especially when γ=50, whereas the HPY conditional calculation was more accurate, though it did slightly overestimate in some cases, in particular when the sample size in the local populations was smaller. Recall that higher values of γ generally correspond to a higher species richness across all populations (i.e., in the metacommunity). Thus, the conditional Gini–Simpson index calculated using the HPY framework is more accurate when there is high species richness in the metacommunity.

The conditional index is also generally more accurate for smaller population sizes. It should be noted that, as the number of samples taken within each population increases, the calculated values of the Gini–Simpson index from all methods are consistently accurate. This shows that the conditional estimates from the HPY model are particularly valuable in samples with a low number of sequences.

It also appears that the HDP model severely overestimates diversity. This appears to be the case when γ is large, and notably, when the local and top‐level discount parameters ($\sigma_{j}$ and α) are large. This is unsurprising, given that the HDP model does not have discount parameters. As a result, the concentration parameters in the HDP tend to be over‐estimated to compensate.

Next, we explore the results of Simpson's index for beta‐diversity. In the HPY model, we estimate this in the same way as for the alpha‐diversity measure of Simpson's index shown above, only we now use Equation (9) for the unconditional index and Equation (10) for the conditional index. Figure S6 shows the error for Simpson's index for beta diversity estimated from the HPY model (unconditional and conditional), the HDP model, and naïvely. There are not many discernible differences between the three estimation methods. This is likely due to the fact that, when calculating the underlying true beta‐diversity values, it is probable that one or both of the proportions from a pair of populations will be zero. Thus, differences in the true beta‐diversity values will be greatly affected by more abundant species, whereas differences in estimated values tend to occur due to differences in rarer species. However, one notable exception is for the HDP model, which once again, substantially overestimates beta diversity when either the discount parameters are large, or the top‐level concentration parameter γ is large.

In the next simulation experiment, we seek to investigate the stability of alpha‐diversity estimation on an initial set of local populations after adding more local populations as input to the HPY sampler. Stability results are shown in Figures S7 (homogeneous case) and S8 (heterogeneous case). These results show good stability of estimates over increasing numbers of local populations included in the model.

In the final simulation experiment, we simulate 20 populations across two separate metacommunities to see if diversity can be compared across metacommunities. The rankings of the Gini–Simpson index across all 20 populations can be seen in Table S1 (Section S3). We also calculate the true and empirical rankings for Gini–Simpson. The rankings for both the empirical and HPY‐C estimators differ slightly from the truth, which is expected since they were both calculated on a subset of the populations. HPY‐C shows perfect concordance with the empirical estimator, which suggests that the sharing of parameters within each metapopulation in calculating HPY‐C is not negatively affecting the ordering of samples based on diversity. We believe that this provides evidence that alpha‐diversity calculations can be compared between datasets.

## 4 Data Analysis Results

We now outline the results of applying the HPY model in the infant gut microbiome study as described in Section 2.5. We also explore the effect of running the HPY versus HDP processes on diversity estimates. For the HPY model, mixing of MCMC chains was very good. Using 4 chains, 300 burn‐in samples, and 1000 MCMC samples, we assessed the potential scale reduction factor for all parameters. For the top‐level parameters, α and β, the $\hat{R}$ values are 1.000094 and 1.001691, respectively. For the local discount parameters $\sigma_{j}$ the mean $\hat{R}$ is 1.0053, and for the local concentration, the mean is 1.0162.

First, we consider the HPY estimated values of Gini–Simpson's index among the different covariate levels.

Table 1 gives the means and standard deviations of Simpson's index from the HPY sampler within each category for breastfeeding status, presence of pets at home, and sex. The HPY conditional estimates are consistently greater than those calculated empirically. Additionally, the HPY unconditional estimates are strikingly larger than estimates from either HPY conditional or empirical. The conditional HDP diversity estimates are typically larger than those from the HPY conditional formula. Though differences between estimates from the various methods are small in magnitude, recall that the motivation of the HPY model is to better capture the tail of the ranked species abundance distribution, that is, the large collection of less abundant microbes.

**TABLE 1:** Gini‐Simpson index (α‐diversity) estimated using HPY (conditional and unconditional), HDP, and empirically in the infant gut microbiome dataset. Each estimate shows the mean (standard deviation) over all subjects in each sub‐category.

		HPY conditional	HDP conditional	HPY unconditional	Empirical
Feeding type	None	0.67565 (0.17126)	0.67568 (0.17123)	0.68575 (0.07713)	0.67548 (0.17133)
	Partial	0.63376 (0.18404)	0.63379 (0.18401)	0.64184 (0.08972)	0.63351 (0.18407)
	Exclusive	0.57202 (0.2048)	0.57202 (0.20473)	0.61153 (0.08747)	0.57163 (0.20485)
Pets at home	No	0.60245 (0.20068)	0.60249 (0.20064)	0.62377 (0.09539)	0.60221 (0.20072)
	Yes	0.61551 (0.19359)	0.61551 (0.19354)	0.64418 (0.08438)	0.61513 (0.19369)
Sex	Female	0.60728 (0.20595)	0.60731 (0.20589)	0.63065 (0.09334)	0.60701 (0.20604)
	Male	0.61067 (0.1883)	0.61067 (0.18827)	0.63727 (0.08776)	0.61032 (0.18834)

Next, we examine the beta‐diversity estimates using Morisita's index; these results are found in Table 2. The mean index estimates are greater across the board for the HPY model compared to the HDP model. While it appears that the HPY and HDP both showed significant differences in beta diversity for within and between pairs in breastfeeding status, the mean empirical estimates do not appear to differ.

**TABLE 2:** Morisita's index (β diversity) estimated using HPY (conditional and unconditional), HDP, and empirically in the infant gut microbiome dataset. Each estimate shows the mean (standard deviation) over all subject pairs in each sub‐category. Bold figures correspond to significant (α=0.05) differences in mean Morisita index between the within‐group and between‐group pairs.

	HPY Conditional		HDP Conditional		Empirical	
	Within	Between	Within	Between	Within	Between
Feeding type	**0.24625 (0.26556)**	**0.25589 (0.26925)**	**0.24621 (0.26553)**	**0.25584 (0.26922)**	0.25406 (0.27445)	0.25110 (0.26582)
Pets at home	0.25329 (0.26953)	0.25092 (0.26616)	0.25324 (0.26950)	0.25088 (0.26612)	0.25284 (0.26956)	0.25169 (0.26893)
Sex	0.25269 (0.26764)	0.25151 (0.26805)	0.25265 (0.26760)	0.25147 (0.26802)	0.25133 (0.26867)	0.25319 (0.26982)

**FIGURE 3:**
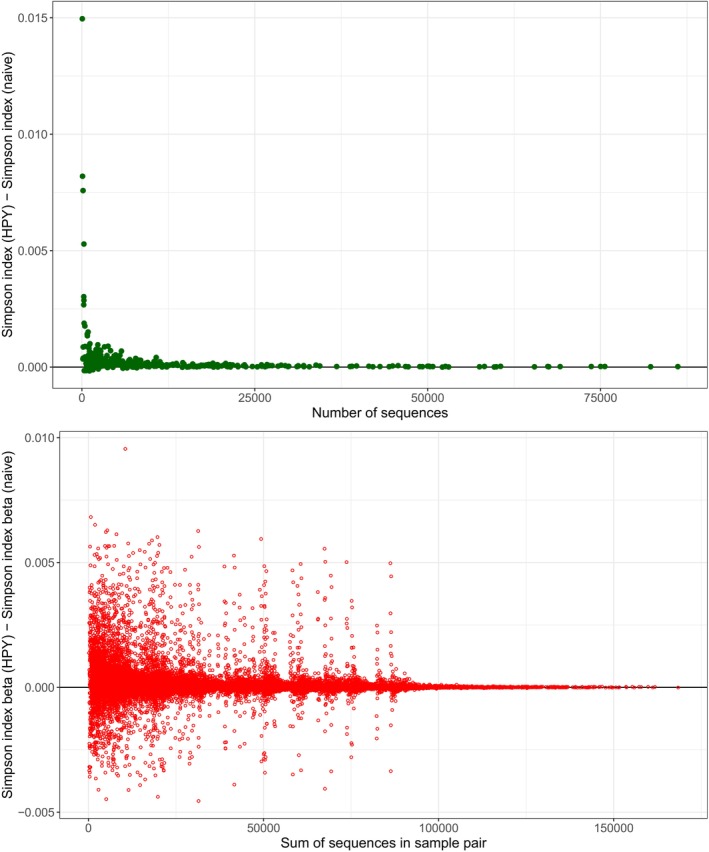
Difference between Gini–Simpson index for **(top)** alpha diversity and **(bottom)** beta diversity calculated using the HPY model and naïvely as a function of the number of sequences in each sample in the infant gut dataset. For the beta‐diversity plot, the sum of the number of sequences for each sample pair is considered.

To further investigate the importance of sampling depth, we look at differences between HPY conditional and empirical estimates of the Gini–Simpson index as a function of sampling depth. This is illustrated in Figure 3. In the alpha‐diversity plot, there are much more pronounced differences in estimates for samples with read depths below 10 000. Likewise, in the beta‐diversity plot, we can see a similar pattern occurring when the sum of read depths between a pair of samples is less than 100 000. This result is consistent with the simulation results presented in Section 3. We see a very similar pattern in Figure S9, which compares HPY and HDP models with respect to read depth. This goes to show that there is a great potential for using the HPY model in diversity estimation, especially in samples with a smaller sequencing depth.

**FIGURE 4:**
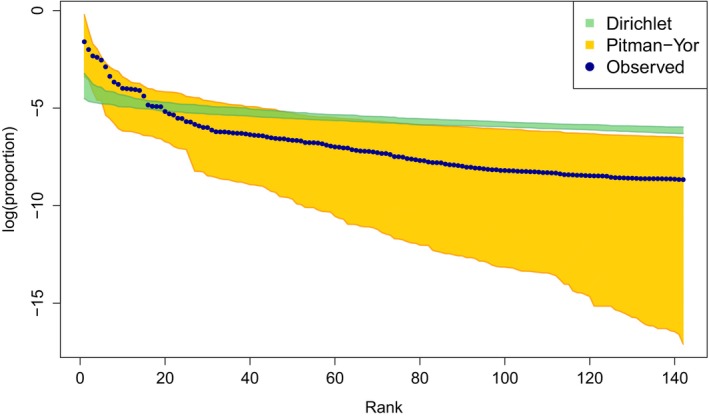
Ranked abundance curves of species. The blue dots represent the true abundances in the infant gut dataset, and the shaded region represents the range of the ranked abundance curves in the simulated data from the HDP and HPY models.

Next, we check the appropriateness of the HPY model on this dataset. To do so, we compare the curve of ranked species abundances (averaged over all subjects in the dataset) to multiple datasets simulated under the HPY model. In each simulation, we ran the HPY process using parameters from one of the MCMC samples from the Gibbs sampler run on the infant gut dataset. We also did the same using a HDP for comparison. Figure 4 shows the range of the ranked species abundance curves for the HPY and Dirichlet models along with the observed species abundance curve from the dataset. It is immediately apparent that the HPY model fits the observed data much better than the hierarchical Dirichlet model. In particular, the HPY model better captures the tail of the distribution, which is unsurprising given the model's ability to handle a power‐law tail. We similarly examined the simulated proportion of nonzero counts among individuals in Figure S10. The proportions of nonzero observations in the HPY model are quite close to the true proportion in the infant gut dataset, on average. The HDP has significantly more nonzero counts than the true dataset, on average. This shows the HPY model can accommodate a large number of zeros despite not having a specific zero‐inflation parameter included in its formulation.

**FIGURE 5:**
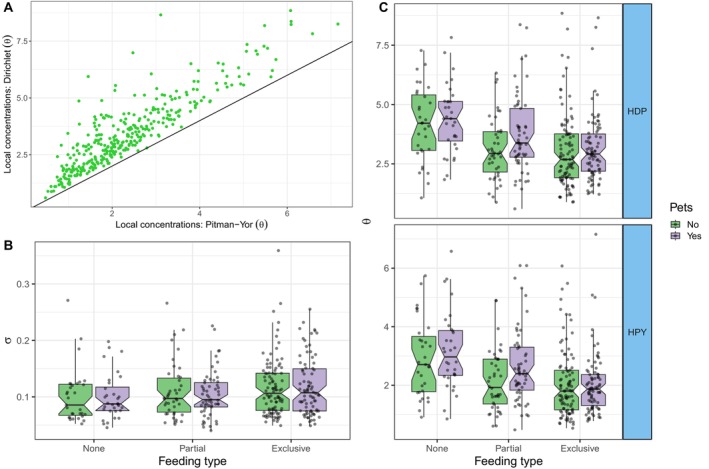
Local parameter results in infant gut data analysis. (A) comparing estimated local concentration parameters between HPY and HDP models. (B) Estimates in the HPY model for local discount ($\sigma_{j}$) (C) Estimates of concentration ($\theta_{j}$) parameters in the HPY and HDP models.

To further investigate the HPY and HDP model results in this dataset, we look at the local‐level parameters estimated within the different covariate groups. First, we compare the local‐level concentration parameters ($\theta_{j}$) between the HDP and HPY models; this can be seen in Figure 5A. The HDP model estimates are invariably greater than those from the HPY model. This is likely due to the absence of a discount parameter $\sigma_{j}$ in the HDP model.

In Figure 5B, we can see the distributions of the estimated local‐level discount parameters ($\sigma_{j}$) among feeding type and pets‐at‐home groups. Note that there is no equivalent parameter in the HDP framework. An ANOVA was run with groups defined by these two covariates and an interaction term, and there was a significant difference in the local‐level discount with respect to feeding type (F=3.180, p=0.0428). This discount parameter increases, on average, when moving between no breastfeeding, partial, and exclusive. Recall that a higher discount parameter corresponds to a departure from the neutrality assumption in the mainland‐island model.

In Figure 5C, we show the estimated local‐level concentration parameters ($\theta_{j}$) in the HPY and HDP models in the feeding type and pets‐at‐home groups. We once again run ANOVAs to compare the average estimates of this parameter in groups defined by feeding type, pets‐at‐home, and their interaction. Both models show significance for the feeding type parameter (HPY: F=17.167, $p=7.96\times 10^{-8}$) and (HDP: F=14.413, $p=9.87\times 10^{-7}$). The mean‐square error of the residuals (MSR) is markedly lower in the HPY model with $MSR_{HPY}=1.370$ and $MSR_{HDP}=2.270$, suggesting less within‐group variation in parameter estimates for the HPY model. Both models estimate a decrease in the concentration parameter, on average, with higher levels of breastfeeding. In performing Tukey's HSD as a post‐hoc test (in order to adjust for family‐wise error rate), we see significant differences between both non‐partial and non‐exclusive feeding groups for both HPY and HDP. However, when considering the exclusive‐partial comparison, the HPY finds a difference (D=−0.3682, p=0.0331) whereas HDP does not (D=−0.3483, p=0.1561).

## 5 Discussion

In this article, we have applied the HPY process to model species abundance distributions in microbiome sequencing data. We have developed a Gibbs sampler to fit the model and shown that the parameters in the HPY process are well estimated in this context. We have additionally provided a general formula for calculation of Hill numbers in the context of the HPY process. Finally, we have derived an estimator of the Gini–Simpson's index (for both alpha‐ and beta diversity) that leverages both HPY estimates from the Gibbs sampler as well as the observed data and inferred ancestral states. This conditional estimator was shown to outperform the unconditional estimator as well as the HDP‐based and naïve estimators in most simulation cases.

In the infant gut dataset, the conditional HPY estimates of the alpha‐diversity Gini–Simpson index were systematically lesser than those from the HDP model, and systematically greater than those from empirical estimates. Unconditional estimates in the HPY model were much greater, on average, than any other method; the unconditional estimates did not perform well in simulations, which further demonstrates the advantages of leveraging the abundance data for estimating diversity rather than simply using the parameter estimates themselves. Furthermore, in comparing HPY and HDP parameter estimates, it appears both in simulations and the infant gut analysis, that the lack of a discount parameter in the HDP model tends to lead to an overestimation of the concentration parameter. We thus have evidence of the appropriateness of favouring the HPY model over the HDP model in real‐world microbial data. The fact that the observed ranked species abundance curve fits the HPY model much better than the HDP model supports this idea. Based on numerical evidence in Figures 4 and S10, we have shown that the HPY model is remarkably attuned to the observed distribution of the infant gut dataset, both in terms of observed proportions, but also in terms of the number of zero counts observed in the data. The main scenario where we would expect worse model performance would be in the case where the true ranked species abundance curve is not of the power‐law type; this could adversely affect diversity estimates. However this effect would be mitigated if the number of organisms sequenced in the local populations is large.

The two procedures related to Figures 4 and S10 could themselves be used as diagnostic tools in practice; for example, re‐simulating values under the fitted model and checking the simulated ranked species abundance curve against the true curve, and checking the proportion of zeros in the re‐simulated data to see concordance with the real data.

The main limitation of the method is the computational inefficiency of the Gibbs sampler. To test the computational scaling we ran the sampler for 1000 iterations in 4 chains, after 300 iterations of burn‐in. We used the infant gut dataset, which had K=901 taxa after preprocessing. For J=50, J=100, and J=356 (the entire dataset), the running times were 8 h 4 min, 14 h 20 min, and 51 h and 17 min, respectively. The number of taxa doesn't affect running time as much as sample size since the number of parameters increases with sample size. For example, running time for J=356 and K=50 is 40 h and 58 min. In particular, resampling the ancestral states $t_{jp}$ is a challenge. Since sequencing depths could be quite high, there could be tens of thousands of ancestral states to update in each iteration of the Gibbs sampler. However, because all individuals of the same species within a population could be considered interchangeable to an extent, we do not have to keep track of the individuals' ancestral states, rather we only need to keep track of the total number of ancestors corresponding to each species. In this case, ancestors can be added or subtracted from each population at a rate that depends on HPY parameters and current ancestor frequencies. Buntine et al. [19] developed a sampler in this style which could be applied instead of the table indicator sampler as described by [14]. Similarly, there have also been developments in variational approximations in Dirichlet mixtures that could be extended for the hierarchical Pitman‐Yor model [27, 28]. Such approximations could greatly reduce the computational times in this model.

An additional limitation is the fact that species defined in next‐generation sequencing methods are not analogous to the typical definition of species in that they comprise nested sets consisting of taxonomically diverse entities. The effect of this is that alpha diversity measures tend to be underestimated, on average.

In this article, when calculating the Gini–Simpson index from the HPY model, we consider the results of sampling the *next two* individuals from the Chinese restaurant representation of the process, after having observed a sample from the target population. However, this is distinct from the “true” diversity in a microbiome population, since the frequencies of the Chinese restaurant process will be different in the true underlying population. To get better estimates of diversity as well as species richness, individuals can be up‐sampled within each population using the Chinese restaurant process. From there, various measures of diversity could be calculated to estimate their true values in the underlying population. This could be useful in particular for diversity measures that would be difficult or impossible to write directly in terms of the HPY parameters.

However, since the Gini–Simpson index is a diversity measure of second order, it is not sensitive to changes in the tail of this distribution. This means that the Gini–Simpson index is not necessarily the best diversity measure for investigating the tail. While it is true that the average differences between the Gini–Simpson index for the different estimation methods are small in magnitude, it should be noted that the differences are systematic. The calculation of this index can be expressed very nicely in terms of the probabilities defined by the Chinese restaurant representation. We believe using this index is a good starting point for demonstrating how diversity measures could be estimated using the HPY process, though future work should focus on alternative metrics, such as the Shannon diversity index, which would be more sensitive to changes in the tail of the ranked species abundance distribution. Though feasible, these estimates will likely prove more challenging to derive in this context compared to Simpson's index. We will consider this in future work.

## 6 Conclusion

We have developed a Gibbs sampler to fit a model based on the HPY process given species abundance data across multiple microbial populations. This process is more appropriate for use on microbiota species abundance data than the previously established HDP due to its ability to accommodate a power‐law tail. Additionally, we have provided new expressions for the Gini–Simpson index measuring alpha diversity and beta diversity in the context of the HPY process and showed their usefulness in simulation and in a data application. In doing so, we also derived a general formula for calculation of the Hill numbers in the HPY framework. We have written an R package that runs the sampler proposed in the article: https://github.com/kevinmcgregor/hpy.

## Author Contributions

K.M. wrote the HPY sampler, derived measures of diversity, performed all simulations and data analyses, and wrote the manuscript. T.P. derived measures of diversity and contributed to manuscript writing. C.Q. conceived the project and contributed to manuscript writing. A.K., E.S., and J.S. were involved in the design, analysis, and publication of the CHILD study data considered in this article.

## Funding

This work was supported by the Natural Sciences and Engineering Research Council of Canada (Grant No. RGPIN‐2021‐03634), the Biotechnology and Biological Sciences Research Council, the Earlham Institute Strategic Programme Grant (Grant Nos. BBX011089/1, BBS/E/ER/230002C), the UK's Biotechnology and Biological Sciences Research Council Grant (Grant Nos. BB/CSP1720/1, BBS/E/T/000PR9818, BBS/E/T/000PR9817, BB/CCG2220/1).

## Conflicts of Interest

The authors declare no conflicts of interest.

## Supporting information




**Figure S1:** Normalized estimation error for top‐level concentration(γ) in the simulation. scenarios. **Figure S2:** Normalized estimation error for top‐level discount(α) in the simulation scenarios. **Figure S3:** Normalized estimation error for local‐level concentrations ($\theta_{j}$) in the simulation scenarios. **Figure S4:** Normalized estimation error for local‐level discounts ($\sigma_{j}$) in the simulation scenarios. **Figure S5:** Estimation error for Simpson's index (alpha diversity) in the simulation scenarios comparing conditional Simpson's index (HPY), unconditional Simpson's index (HPY‐U) and naïve estimates. **Figure S6:** Estimation error for Simpson's index (beta diversity) in the simulation scenarios. **Figure S7:** Stability of the Gini–Simpson index estimated under the HPY model (homogeneous populations) in 10 local populations when additional populations are added to the dataset. **Figure S8:** Stability of the Gini–Simpson index estimated under the HPY model (heterogeneous populations) in 10 local populations when additional populations are added to the dataset. **Figure S9:** Difference in HPY and HDP estimates of Simpson's index for (**top**) alpha diversity and (**bottom**) beta diversity calculated as a function of the number of sequences in each sample in the infant gut dataset. For the beta‐diversity plot, the sum of the number of sequences for each sample pair is considered. **Figure S10:** Difference in the proportion of simulated nonzero elements in simulated data vs. true infant gut data. The data was simulated under parameters estimated from the infant gut dataset. **Table S1:** Rankings of true and estimated Gini–Simpson index (HPY‐C and empirical) across 20 local populations drawn from two separate metacommunities.

## Data Availability

The data that support the findings of this study are available on request from the corresponding author. The data are not publicly available due to privacy or ethical restrictions.
